# Supplementary material for: Synthetic lethality between TP53 and ENDOD1
Source: Nat Commun. 2022 May 23;13:2861. doi: 10.1038/s41467-022-30311-w (PMC9126970; doi:10.1038/s41467-022-30311-w)
Supplement: Supplementary file 3 — Reporting Summary [file 41467_2022_30311_MOESM3_ESM.pdf]

nature portfolio

Corresponding author(s): Antony M. Carr
Cong Liu

Last updated by author(s): Jan 18, 2022

Reporting Summary

Nature Portfolio wishes to improve the reproducibility of the work that we publish. This form provides structure for consistency and transparency in reporting. For further information on Nature Portfolio policies, see our Editorial Policies and the Editorial Policy Checklist.

Statistics

For all statistical analyses, confirm that the following items are present in the figure legend, table legend, main text, or Methods section.

n/a	Confirmed	
☐	☒	The exact sample size (*n*) for each experimental group/condition, given as a discrete number and unit of measurement
☐	☒	A statement on whether measurements were taken from distinct samples or whether the same sample was measured repeatedly
☐	☒	The statistical test(s) used AND whether they are one- or two-sided<br>*Only common tests should be described solely by name; describe more complex techniques in the Methods section.*
☒	☐	A description of all covariates tested
☒	☐	A description of any assumptions or corrections, such as tests of normality and adjustment for multiple comparisons
☐	☒	A full description of the statistical parameters including central tendency (e.g. means) or other basic estimates (e.g. regression coefficient) AND variation (e.g. standard deviation) or associated estimates of uncertainty (e.g. confidence intervals)
☐	☒	For null hypothesis testing, the test statistic (e.g. *F*, *t*, *r*) with confidence intervals, effect sizes, degrees of freedom and *P* value noted<br>*Give P values as exact values whenever suitable.*
☒	☐	For Bayesian analysis, information on the choice of priors and Markov chain Monte Carlo settings
☒	☐	For hierarchical and complex designs, identification of the appropriate level for tests and full reporting of outcomes
☒	☐	Estimates of effect sizes (e.g. Cohen's *d*, Pearson's *r*), indicating how they were calculated

Our web collection on statistics for biologists contains articles on many of the points above.

Software and code

Policy information about availability of computer code

Data collection	No custom software has been used.
Data analysis	No custom software has been used. We describe software used for data analysis in Methods. Mass spectrometry: Maxquant search engine (v.1.6.6.0). Image processing/quantification: Image J (v.1.52); Image Pro Plus (v.6.0). Flow cytometry: ModFit (v.5.0) and Flow Jo (v.10). Cardiac analysis: Vevostrain. Statistical analysis: GraphPad Prism (v.6); SPSS (v.16.0). Non-parametric Kruskal tests, Python (v.3.8) scipy.stats

For manuscripts utilizing custom algorithms or software that are central to the research but not yet described in published literature, software must be made available to editors and reviewers. We strongly encourage code deposition in a community repository (e.g. GitHub). See the Nature Portfolio guidelines for submitting code & software for further information.

Data

Policy information about availability of data

All manuscripts must include a data availability statement. This statement should provide the following information, where applicable:

- Accession codes, unique identifiers, or web links for publicly available datasets
- A description of any restrictions on data availability
- For clinical datasets or third party data, please ensure that the statement adheres to our policy

SwissProt (2019) was used to identify peptides. The remaining data that support the findings of this study are available from the corresponding authors upon reasonable request. All the data are standard lab data collected for graphs, or microscope images.

Field-specific reporting

Please select the one below that is the best fit for your research. If you are not sure, read the appropriate sections before making your selection.

☒ Life sciences ☐ Behavioural & social sciences ☐ Ecological, evolutionary & environmental sciences

For a reference copy of the document with all sections, see nature.com/documents/nr-reporting-summary-flat.pdf

Life sciences study design

All studies must disclose on these points even when the disclosure is negative.

Sample size	For quantitative staining >150 cells were counted to provide robust statistical analysis (p-value is less than alpha). Comet assay: 150 cells from 3 independent experiments were measured (Olive PL, Banath JP (2006) The comet assay: a method to measure DNA damage in individual cells. Nat Protoc 1(1):23–29). For in vivo studies, the sample size was determined using a power calculation for p<0.05, power >0.8 based on informed estimates by the statistic expert on the animal ethics committee. Preliminary experiments (survival, pathology & body weghts) using in vivo siWdr70 data helped estimate the sample size for the side-effect experiments of siEndod1 (Fig 6a-e).
Data exclusions	One subject in simWdr70 group (Figure 7) was dead before termination of the experiment. Data of body weight for this mouse was excluded in Figure 7a. No data in other experiments has been excluded.
Replication	All experiments are reproducible and have been replicated at least 3 times. Our scientific education has always been experiments need to be reproducible and that three replicates is the minimum to ensure this.
Randomization	For animal experiment, animals were randomized using random number table into control and treatment groups. This is described in Methods. All other experiments are cell culture based and required no randomization. For example, culture dishes after splitting cells were assumed to be identical for the same cell line and were treated, or not, by simply taking the first dish of cell line x.
Blinding	Based on our standard laboratory practice, cell biology assays were not blinded to investigators performing data collection and analysis. The animal experiments in Figure 6b-e and Supplementary Figure 8b-c were blinded to data collectors and investigators.

Behavioural & social sciences study design

All studies must disclose on these points even when the disclosure is negative.

Study description	*Briefly describe the study type including whether data are quantitative, qualitative, or mixed-methods (e.g. qualitative cross-sectional, quantitative experimental, mixed-methods case study).*
Research sample	*State the research sample (e.g. Harvard university undergraduates, villagers in rural India) and provide relevant demographic information (e.g. age, sex) and indicate whether the sample is representative. Provide a rationale for the study sample chosen. For studies involving existing datasets, please describe the dataset and source.*
Sampling strategy	*Describe the sampling procedure (e.g. random, snowball, stratified, convenience). Describe the statistical methods that were used to predetermine sample size OR if no sample-size calculation was performed, describe how sample sizes were chosen and provide a rationale for why these sample sizes are sufficient. For qualitative data, please indicate whether data saturation was considered, and what criteria were used to decide that no further sampling was needed.*
Data collection	*Provide details about the data collection procedure, including the instruments or devices used to record the data (e.g. pen and paper, computer, eye tracker, video or audio equipment) whether anyone was present besides the participant(s) and the researcher, and whether the researcher was blind to experimental condition and/or the study hypothesis during data collection.*
Timing	*Indicate the start and stop dates of data collection. If there is a gap between collection periods, state the dates for each sample cohort.*
Data exclusions	*If no data were excluded from the analyses, state so OR if data were excluded, provide the exact number of exclusions and the rationale behind them, indicating whether exclusion criteria were pre-established.*
Non-participation	*State how many participants dropped out/declined participation and the reason(s) given OR provide response rate OR state that no participants dropped out/declined participation.*
Randomization	*If participants were not allocated into experimental groups, state so OR describe how participants were allocated to groups, and if allocation was not random, describe how covariates were controlled.*

Ecological, evolutionary & environmental sciences study design

All studies must disclose on these points even when the disclosure is negative.

Study description	*Briefly describe the study. For quantitative data include treatment factors and interactions, design structure (e.g. factorial, nested, hierarchical), nature and number of experimental units and replicates.*
Research sample	*Describe the research sample (e.g. a group of tagged Passer domesticus, all Stenocereus thurberi within Organ Pipe Cactus National Monument), and provide a rationale for the sample choice. When relevant, describe the organism taxa, source, sex, age range and any manipulations. State what population the sample is meant to represent when applicable. For studies involving existing datasets, describe the data and its source.*
Sampling strategy	*Note the sampling procedure. Describe the statistical methods that were used to predetermine sample size OR if no sample-size calculation was performed, describe how sample sizes were chosen and provide a rationale for why these sample sizes are sufficient.*
Data collection	*Describe the data collection procedure, including who recorded the data and how.*
Timing and spatial scale	*Indicate the start and stop dates of data collection, noting the frequency and periodicity of sampling and providing a rationale for these choices. If there is a gap between collection periods, state the dates for each sample cohort. Specify the spatial scale from which the data are taken*
Data exclusions	*If no data were excluded from the analyses, state so OR if data were excluded, describe the exclusions and the rationale behind them, indicating whether exclusion criteria were pre-established.*
Reproducibility	*Describe the measures taken to verify the reproducibility of experimental findings. For each experiment, note whether any attempts to repeat the experiment failed OR state that all attempts to repeat the experiment were successful.*
Randomization	*Describe how samples/organisms/participants were allocated into groups. If allocation was not random, describe how covariates were controlled. If this is not relevant to your study, explain why.*
Blinding	*Describe the extent of blinding used during data acquisition and analysis. If blinding was not possible, describe why OR explain why blinding was not relevant to your study.*

Did the study involve field work? ☐ Yes ☐ No

Field work, collection and transport

Field conditions	*Describe the study conditions for field work, providing relevant parameters (e.g. temperature, rainfall).*
Location	*State the location of the sampling or experiment, providing relevant parameters (e.g. latitude and longitude, elevation, water depth).*
Access & import/export	*Describe the efforts you have made to access habitats and to collect and import/export your samples in a responsible manner and in compliance with local, national and international laws, noting any permits that were obtained (give the name of the issuing authority, the date of issue, and any identifying information).*
Disturbance	*Describe any disturbance caused by the study and how it was minimized.*

Reporting for specific materials, systems and methods

We require information from authors about some types of materials, experimental systems and methods used in many studies. Here, indicate whether each material, system or method listed is relevant to your study. If you are not sure if a list item applies to your research, read the appropriate section before selecting a response.

Materials & experimental systems

n/a	Involved in the study	
☐	☒	Antibodies
☐	☒	Eukaryotic cell lines
☒	☐	Palaeontology and archaeology
☐	☒	Animals and other organisms
☒	☐	Human research participants
☒	☐	Clinical data
☒	☐	Dual use research of concern

Methods

n/a	Involved in the study	
☒	☐	ChIP-seq
☐	☒	Flow cytometry
☒	☐	MRI-based neuroimaging

Antibodies

Antibodies used

Antibody information is described in Supplementary Table S4 and is reproduced here.
Rabbit anti-phosphor-Serine 33, RPA32 NOVUS, NB100-544
Mouse anti-α-Tubulin Sigma, T6074
Mouse anti-phospho Serine 139, gH2AX Millipore, 05-636
Rabbit anti-53BP1 Bethyl, A300-272A
Rabbit anti-phosphor-Serine 1524, BRCA1 Bethyl, A300-001A
Rabbit anti-BRCA1 Huabio, HA500015
Mouse anti-PAR Abcam, ab14459
Rabbit anti-MRE11 CST, 4895S,
Rabbit anti-CTIP NOVUS, NB100-79810
Rabbit anti-BLM Bethyl, A300-110A-2
Rabbit anti-phospho-Serine 343, NBS1 Abcam, ab109453
Rabbit anti-NBS1 Huabio, ET1610-26
Rabbit anti-XRCC1 Huabio, ET1704-01
Mouse anti-BrdU Roche, 11585860001
Rabbit anti-FANCD2 NOVUS, NBP2-57171
Rabbit anti-Flag Custom made
Rabbit anti-p53 HUABIO, ET1601-13
Rabbit anti-PARP1 HUABIO, ER1802-67
Rabbit anti-PARP2 HUABIO, ET7108-05
Rabbit anti-PARP3 NOVUS, NBP2-49523
Mouse anti-H3 Millipore, 05-1341
Rabbit anti-ENDOD1 ABcam, ab121293
Rabbit anti-ENDOD1 ABclonal, A16502
HRP-conjugated anti-mouse IgG DAKO, P0260
HRP-conjugated anti-rabbit IgG DAKO, P0448
FITC-conjugated anti-mouse IgG Sigma, F0257
CY3- conjugated anti- rabbit IgG Sigma, C2306
B220 (Efluor 450) Invitrogen, 48-0452-82
CD3e (PE-cy7) Invitrogen, 25-0031-82
Ly-6G/Gr-1 (APC) Invitrogen, 17-9668-82
F480 (FITC) Biolegend, B257636
CD11b (APC) Biolegend, B261578
NK1.1 (PE-cy7) Invitrogen, 25-5941-82
Lineage (Efluor 450) Invitrogen, 88-7772-72
Sca-1 (PE) Invitrogen, 12-5981-83
c-Kit (APC) invitrogen, 17-1171-82

Validation

All commercial antibodies are validated for use in/on human cells/cell extracts by the respective company (details available on company websites).
Rabbit anti-phosphor-Serine 33-RPA2, validated by manufacturer for WB, IF;
Mouse anti-α-Tubulin, validated by manufacturer for WB, IF;
Mouse anti-phospho Serine 139-gamaH2AX, validated by manufacturer for WB ,IF
Rabbit anti-53BP1, validated by manufacturer for WB, IP, IHC;
Rabbit anti-phosphor-Serine 1524-BRCA1, validated by manufacturer for WB
Rabbit anti-BRCA1, validated by manufacturer for WB.
Mouse anti-PAR, validated by citation: doi: 10.1186/s12885-016-2656-8
Rabbit anti-MRE11, validated by manufacturer for WB, ICC/IF, IP
Rabbit anti-CTIP, validated by manufacturer for WB, ICC/IF, IP
Rabbit anti-BLM, validated by citation for IF: doi: 10.1038/s41467-019-10179-z
Rabbit anti-phospho-Serine 343-NBS1, validated by manufacturer for Dot blot, WB, ICC
Rabbit anti-NBS1, validated by manufacturer for WB, ICC/IF,IP,FC,IHC-P, ChIP
Rabbit anti-XRCC1, validated by manufacturer for WB
Mouse anti-BrdU, validated by manufacturer for Elisa,ICC
Rabbit anti-FANCD2, validated by manufacturer for ICC/IF
Rabbit anti-Flag, validated by our previous publication for IP, WB: doi: 10.1002/hep.30571
Rabbit anti-p53, validated by manufacturer for WB, ICC/IF,IHC-P.
Rabbit anti-3meH3K27, validated by manufacturer for WB, ICC/IF
Rabbit anti-PARP1, validated by manufacturer for WB, ICC, FC
Rabbit anti-PARP2, validated by manufacturer for WB, FC
Rabbit anti-PARP3, validated by manufacturer for WB, IHC, IHC-P
Mouse anti-H3, validated by manufacturer for WB, IP, FC, Elisa
Rabbit anti-ENDOD1(ABcam), validated by manufacturer for ICC/IF, IHC-P; validated in this study for WB.
Rabbit anti-ENDOD1(ABclonal), validated in this study for IF (Figure 1B).
Armenian hamster anti-B220, Armenian hamster anti-CD3e, Rat anti-Ly-6G/Gr-1, Mouse anti- NK1.1, Rat anti-Lineage, Rat anti- Sca-1, Rat anti- c-Kit, validated by manufacturer for FC.
In addition we performed our own validation on the following antibodies: anti-ENDOD1 by siRNA in Figure 1d, S1b, S3a and S10d; anti-PARP1/2/3 by siRNA in Figure S10a; anti-CHK1/BRCA2/ARID1A/MRE11 by siRNA in Figure S10b; anti-p53 by siRNA in Figure 5c, 5h and 10b.

Eukaryotic cell lines

Policy information about cell lines

Cell line source(s)	Cell lines were obtained either from the National Infrastructure of Cell Line Resource, Shanghai: (RPE1, MRC-5, IMR-90, FHs 74, HFL1, WISH, MCF10A, HepG2, HepG2.2.15, MHCC97L, Hep3B, SMMC7721, SKOV-3, OVCAR-8, C33A, Hela, NCI-H1299, NCI-H1975, A549, SW480, HO8910, HO8910-PM, COLO-320DM, T43, MDA-MB-468, MDA-MB-231, MDA-MB-361, HCC1937, MCF7, U2OS, SH-SY5Y, K562, HL-60, Raji) or from iCell Bioscience Inc, Shanghai (L02, GES-1).
Authentication	All the cell lines were authenticated by providers by STR profile. In addition RPE1, MDA-MB-468, K562, OVCAR-8, HCC1937, HepG2 and HCT116 cells were re-validated by LGT standards (ATCC accredited) by STR profile.
Mycoplasma contamination	All cell lines were tested negative for mycoplasma contamination.
Commonly misidentified lines (See ICLAC register)	None of the cell lines used in this study is identified in the ICLAC list.

Palaeontology and Archaeology

Specimen provenance	*Provide provenance information for specimens and describe permits that were obtained for the work (including the name of the issuing authority, the date of issue, and any identifying information). Permits should encompass collection and, where applicable, export.*
Specimen deposition	*Indicate where the specimens have been deposited to permit free access by other researchers.*
Dating methods	*If new dates are provided, describe how they were obtained (e.g. collection, storage, sample pretreatment and measurement), where they were obtained (i.e. lab name), the calibration program and the protocol for quality assurance OR state that no new dates are provided.*

☐ Tick this box to confirm that the raw and calibrated dates are available in the paper or in Supplementary Information.

Ethics oversight	*Identify the organization(s) that approved or provided guidance on the study protocol, OR state that no ethical approval or guidance was required and explain why not.*

Note that full information on the approval of the study protocol must also be provided in the manuscript.

Animals and other organisms

Policy information about studies involving animals; ARRIVE guidelines recommended for reporting animal research

Laboratory animals	Female and male C57BL/6 (aged 6-8 weeks and sexually randomized) and female BALB/c nu/nu (aged 6-8 weeks) mice were used in Figure 7 and Supplementary 8. Animals were sourced from Gempharmatech co., Ltd.
Wild animals	No wild animals were used in the study.
Field-collected samples	No field collected samples were used in the study.
Ethics oversight	The animal experiments in this study were registered and approved by the Medical Ethical Committee of the West China Second University Hospital of Sichuan University on 8th June, 2018 (approval Reference number, Medical Research 2018 (015).

Note that full information on the approval of the study protocol must also be provided in the manuscript.

Human research participants

Policy information about studies involving human research participants

Population characteristics	*Describe the covariate-relevant population characteristics of the human research participants (e.g. age, gender, genotypic information, past and current diagnosis and treatment categories). If you filled out the behavioural & social sciences study design questions and have nothing to add here, write "See above."*
Recruitment	*Describe how participants were recruited. Outline any potential self-selection bias or other biases that may be present and how these are likely to impact results.*
Ethics oversight	*Identify the organization(s) that approved the study protocol.*

Note that full information on the approval of the study protocol must also be provided in the manuscript.

Clinical data

Policy information about clinical studies

All manuscripts should comply with the ICMJE guidelines for publication of clinical research and a completed CONSORT checklist must be included with all submissions.

Clinical trial registration	*Provide the trial registration number from ClinicalTrials.gov or an equivalent agency.*
Study protocol	*Note where the full trial protocol can be accessed OR if not available, explain why.*
Data collection	*Describe the settings and locales of data collection, noting the time periods of recruitment and data collection.*
Outcomes	*Describe how you pre-defined primary and secondary outcome measures and how you assessed these measures.*

Dual use research of concern

Policy information about dual use research of concern

Hazards

Could the accidental, deliberate or reckless misuse of agents or technologies generated in the work, or the application of information presented in the manuscript, pose a threat to:

No	Yes	
☐	☐	Public health
☐	☐	National security
☐	☐	Crops and/or livestock
☐	☐	Ecosystems
☐	☐	Any other significant area

Experiments of concern

Does the work involve any of these experiments of concern:

No	Yes	
☐	☐	Demonstrate how to render a vaccine ineffective
☐	☐	Confer resistance to therapeutically useful antibiotics or antiviral agents
☐	☐	Enhance the virulence of a pathogen or render a nonpathogen virulent
☐	☐	Increase transmissibility of a pathogen
☐	☐	Alter the host range of a pathogen
☐	☐	Enable evasion of diagnostic/detection modalities
☐	☐	Enable the weaponization of a biological agent or toxin
☐	☐	Any other potentially harmful combination of experiments and agents

ChIP-seq

Data deposition

☐ Confirm that both raw and final processed data have been deposited in a public database such as GEO.

☐ Confirm that you have deposited or provided access to graph files (e.g. BED files) for the called peaks.

Data access links<br>*May remain private before publication.*	*For "Initial submission" or "Revised version" documents, provide reviewer access links. For your "Final submission" document, provide a link to the deposited data.*
Files in database submission	*Provide a list of all files available in the database submission.*
Genome browser session<br>(e.g. UCSC)	*Provide a link to an anonymized genome browser session for "Initial submission" and "Revised version" documents only, to enable peer review. Write "no longer applicable" for "Final submission" documents.*

Methodology

Replicates	*Describe the experimental replicates, specifying number, type and replicate agreement.*
Sequencing depth	*Describe the sequencing depth for each experiment, providing the total number of reads, uniquely mapped reads, length of reads and*

Sequencing depth	*whether they were paired- or single-end.*
Antibodies	*Describe the antibodies used for the ChIP-seq experiments; as applicable, provide supplier name, catalog number, clone name, and lot number.*
Peak calling parameters	*Specify the command line program and parameters used for read mapping and peak calling, including the ChIP, control and index files used.*
Data quality	*Describe the methods used to ensure data quality in full detail, including how many peaks are at FDR 5% and above 5-fold enrichment.*
Software	*Describe the software used to collect and analyze the ChIP-seq data. For custom code that has been deposited into a community repository, provide accession details.*

Flow Cytometry

Plots

Confirm that:

☒ The axis labels state the marker and fluorochrome used (e.g. CD4-FITC).

☒ The axis scales are clearly visible. Include numbers along axes only for bottom left plot of group (a 'group' is an analysis of identical markers).

☒ All plots are contour plots with outliers or pseudocolor plots.

☒ A numerical value for number of cells or percentage (with statistics) is provided.

Methodology

Sample preparation	1. Mouse whole blood were treated with chilled RBC buffer (15 mM NH4Cl, 1 mM KHCO3, 0.1 mM EDTA, pH 7.1-7.4) for 10 min to cause lysis. Sample was then centrifuged for 5 min at 400 x g and cells (1-2 x 10^6 cells/sample) and resuspended in 100 µl PBS, followed by incubation with the appropriate dilution of fluorescent antibody conjugates (CD3e (PE-cy7), NK1.1 (PE-cy7), Ly-6G/Gr-1 (APC), CD11b (APC), F480 (FITC), B220 (eFEfluor 450)) for 30 min at room temperature or 45 min on ice. 2. For bone marrow hematopoietic stem cells (HSC) analysis, a single cell suspension was obtained by flushing bone marrow cells with PBS containing 2% FBS, followed by incubating with marker antibody (Lin(Pacific Blue),c-Kit (APC), Sca-1 (PE)). 3. For bone marrow myeloid and lymphoid populations analysis, a single cell suspension was obtained by flushing bone marrow cells with PBS containing 2% FBS, followed by incubating with marker antibody (Ly-6G/Gr-1 (APC), CD11b (APC), F4/80 (FITC), CD3e (PE-cy7), B220 (Efluor eFluor 450) NK1.1 (PE-cy7)). 4. Cell cycle analysis was performed using 3 x 10^4 trypsin-dissociated cells. After rinsing in PBS two times cells were fixated with 7570% ethanol overnight and stained with PBS-PI (50 µg/ml) for 20 min.
Instrument	BD FACSCalibur, Beckman Cytoflex S
Software	ModFit (v.5.0) and Flow Jo (v.10)
Cell population abundance	After FSC/SSC gating 10,000 - 20,000 cells or events was collected for all experiments. For FACS experiments, cell populations were approximately 98% pure which was determined by post-sort analysis.
Gating strategy	FSC and SSC were used to remove cell debris and dead cells (small FSC v SSC) and aggregated cells or large clumps (large FSC or SSC). Cells were considered as live cells after FSC/SSC gating and then used in fluorescent histograms.<br>Sorted cells were defined as the following: T cells (CD3e+), NK cells (NK1.1+), granulocyte (Gr-1+), macrophage (F480+ and CD11b+), hematopoietic stem cells (Sca1+ and c-Kit+). The border between negative and positive was determined by an isotype-matched control antibody.<br>For cell cycle analysis, the G1 and G2 phase were determined by MODFIT software.

☒ Tick this box to confirm that a figure exemplifying the gating strategy is provided in the Supplementary Information.

Magnetic resonance imaging

Experimental design

Design type	*Indicate task or resting state; event-related or block design.*
Design specifications	*Specify the number of blocks, trials or experimental units per session and/or subject, and specify the length of each trial or block (if trials are blocked) and interval between trials.*
Behavioral performance measures	*State number and/or type of variables recorded (e.g. correct button press, response time) and what statistics were used to establish that the subjects were performing the task as expected (e.g. mean, range, and/or standard deviation across subjects).*

Acquisition

Imaging type(s) | *Specify: functional, structural, diffusion, perfusion.*

Field strength | *Specify in Tesla*

Sequence & imaging parameters | *Specify the pulse sequence type (gradient echo, spin echo, etc.), imaging type (EPI, spiral, etc.), field of view, matrix size, slice thickness, orientation and TE/TR/flip angle.*

Area of acquisition | *State whether a whole brain scan was used OR define the area of acquisition, describing how the region was determined.*

Diffusion MRI ☐ Used ☐ Not used

Preprocessing

Preprocessing software | *Provide detail on software version and revision number and on specific parameters (model/functions, brain extraction, segmentation, smoothing kernel size, etc.).*

Normalization | *If data were normalized/standardized, describe the approach(es): specify linear or non-linear and define image types used for transformation OR indicate that data were not normalized and explain rationale for lack of normalization.*

Normalization template | *Describe the template used for normalization/transformation, specifying subject space or group standardized space (e.g. original Talairach, MNI305, ICBM152) OR indicate that the data were not normalized.*

Noise and artifact removal | *Describe your procedure(s) for artifact and structured noise removal, specifying motion parameters, tissue signals and physiological signals (heart rate, respiration).*

Volume censoring | *Define your software and/or method and criteria for volume censoring, and state the extent of such censoring.*

Statistical modeling & inference

Model type and settings | *Specify type (mass univariate, multivariate, RSA, predictive, etc.) and describe essential details of the model at the first and second levels (e.g. fixed, random or mixed effects; drift or auto-correlation).*

Effect(s) tested | *Define precise effect in terms of the task or stimulus conditions instead of psychological concepts and indicate whether ANOVA or factorial designs were used.*

Specify type of analysis: ☐ Whole brain ☐ ROI-based ☐ Both

Statistic type for inference (See Eklund et al. 2016) | *Specify voxel-wise or cluster-wise and report all relevant parameters for cluster-wise methods.*

Correction | *Describe the type of correction and how it is obtained for multiple comparisons (e.g. FWE, FDR, permutation or Monte Carlo).*

Models & analysis

n/a	Involved in the study
☐	☐ Functional and/or effective connectivity
☐	☐ Graph analysis
☐	☐ Multivariate modeling or predictive analysis

Functional and/or effective connectivity | *Report the measures of dependence used and the model details (e.g. Pearson correlation, partial correlation, mutual information).*

Graph analysis | *Report the dependent variable and connectivity measure, specifying weighted graph or binarized graph, subject- or group-level, and the global and/or node summaries used (e.g. clustering coefficient, efficiency, etc.).*

Multivariate modeling and predictive analysis | *Specify independent variables, features extraction and dimension reduction, model, training and evaluation metrics.*
